# Supplementary figures and images for: Ninein, a candidate gene for ethanol anxiolysis, shows complex exon-specific expression and alternative splicing differences between C57BL/6J and DBA/2J mice
Source: Front Genet. 2024 Sep 11;15:1455616. doi: 10.3389/fgene.2024.1455616 (PMC11422218; doi:10.3389/fgene.2024.1455616)

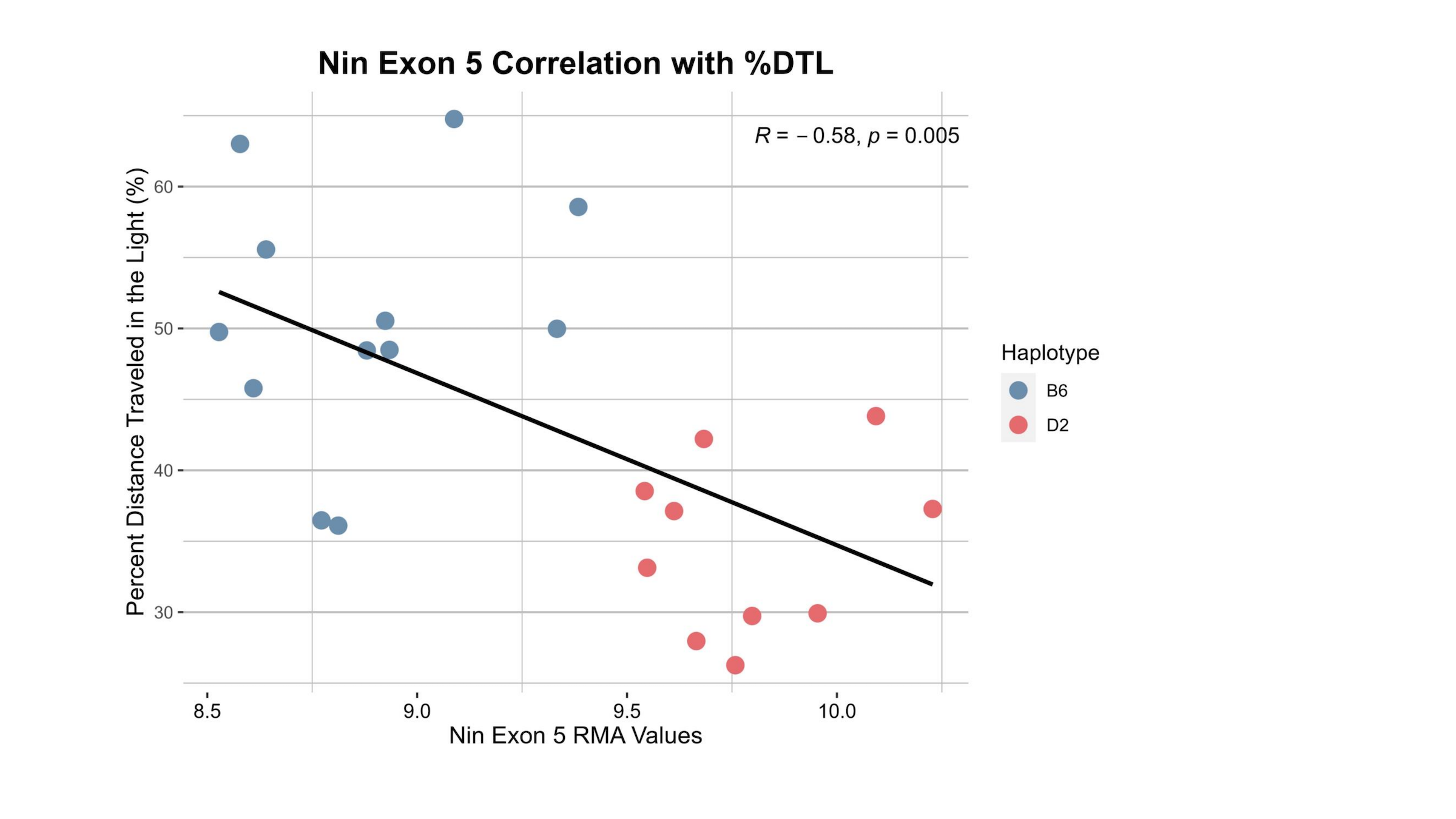

Supplement: Supplementary file 1 [file Image1.TIFF]
