# Supplementary material for: Ninein, a candidate gene for ethanol anxiolysis, shows complex exon-specific expression and alternative splicing differences between C57BL/6J and DBA/2J mice
Source: Front Genet. 2024 Sep 11;15:1455616. doi: 10.3389/fgene.2024.1455616 (PMC11422218; doi:10.3389/fgene.2024.1455616)
Supplement: Supplementary file 3 [file Table1.DOCX]

| **Primer Target** | **Forward Primer (5’ to 3’)** | **Reverse Primer (5’ to 3’)** | **Product Length** |
| --- | --- | --- | --- |
| Nin  A5’S Ex28 | ACAGGTGAGACTGGACGAGAA | AGCAAAGCCTGTGGTGTGTT | 237 |
| Nin  Ex29 (+) | AGTGGTTGGAAGCAGCCGA | ACCATCGGACAGGCTTGCT | 142 |
| Nin  Ex18 (-) | GAGGGAAGATATGAATCTGAAAAGC | TTGGGAGTTCTTTTGGCTGAG | 276 |
| Nin  3’ UTR | AAACCAACACACCACAGGGAA | CTTTGGTGTGCATCCTTTGGG | 366 |
| Nin  Ex32-33 | ACTAAAGCTGGTGAAGAGACTT | GACGATGGTACTGAGGCTGG | 214 |
| Ublcp1 | TCCTGGTGCTGGATGTTGAC | TCACGCCCAGCTCTTTCATT | 183 |
| Ppp2r2a | ATCTCTCACCCTTGCCCTTT | CCCATTTTGTGTGCTTTCGT | 79 |
| Stab2b | ACCTCACCCCTCTGGTTAGT | ACAGATAGGGCAGAACAGGC | 146 |
| Sort1 | ATCTCTCACCCTTGCCCTTT | GAAGGCTGCACTCGTTCTTG | 202 |

**Supplementary Table 1.** Primers used for quantitative reverse-transcriptase PCR (qRT-PCR). (+)Product size confirms Ex29 is present. (-)Product size confirms Ex18 is not present. *Ublcp1, Ppp2r2a, and Sort1* were used as reference genes to calculate relative expression. *Stab2b* was used as a strain control.
