# Supplementary material for: Ninein, a candidate gene for ethanol anxiolysis, shows complex exon-specific expression and alternative splicing differences between C57BL/6J and DBA/2J mice
Source: Front Genet. 2024 Sep 11;15:1455616. doi: 10.3389/fgene.2024.1455616 (PMC11422218; doi:10.3389/fgene.2024.1455616)
Supplement: Supplementary file 5 [file Table2.DOCX]

| **Sample** | **RIN Value** | **Uniquely Mapped Alignment %** | **Assigned %** |
| --- | --- | --- | --- |
| B14 N | 9 | 92.90% | 74.40% |
| B21 N | 9.1 | 91.60% | 74.90% |
| B24 N | 9.3 | 90.20% | 75.40% |
| B31 N | 9.1 | 90.90% | 74.60% |
| B32 N | 9.2 | 92.00% | 75.90% |
| D11 N | 9.3 | 92.50% | 75.00% |
| D13 N | 9.3 | 92.40% | 73.60% |
| D22 N | 9.1 | 92.40% | 74.40% |
| D32 N | 9.4 | 91.60% | 74.20% |
| D34 N | 9 | 91.20% | 74.60% |

**Supplementary Table 2.** RNA-sequencing information, quality control metrics, alignment percentages, and count generation analytics.
